# Development of Cardiac Computed Tomography for Evaluation of Aortic Valve Stenosis

**DOI:** 10.3390/tomography11060062

**Published:** 2025-05-28

**Authors:** Hiroyuki Takaoka, Haruka Sasaki, Joji Ota, Yoshitada Noguchi, Moe Matsumoto, Kazuki Yoshida, Katsuya Suzuki, Shuhei Aoki, Satomi Yashima, Makiko Kinoshita, Noriko Suzuki-Eguchi, Yoshio Kobayashi

**Affiliations:** 1 Department of Cardiovascular Medicine, Chiba University Graduate School of Medicine, Chiba 260-8677, Japan; 2 Department of Radiology, Chiba University Hospital, Chiba 260-8677, Japan

**Keywords:** aortic stenosis, computed tomography, coronary artery

## Abstract

Aortic valve stenosis (AS) is a valvular heart disease that imposes a high afterload on the left ventricle (LV) due to restricted opening of the aortic valve, resulting in LV hypertrophy. Severe AS can lead to syncope, angina pectoris, and heart failure. The number of patients with AS has been increasing due to aging populations, the growing prevalence of lifestyle-related diseases, and advances in diagnostic technologies. Therefore, accurate diagnosis and appropriate treatment of AS are essential. In recent years, transcatheter aortic valve implantation (TAVI) has become feasible, and the number of procedures has rapidly increased, particularly among elderly patients. As treatment options for AS expand and diversify, detailed pre-procedural evaluation has become increasingly important. In particular, diagnostic imaging modalities such as computed tomography (CT) have advanced significantly, with notable improvements in image quality. With recent advancements in CT technology—such as increased detector rows, faster gantry rotation speeds, new image reconstruction methods, and the introduction of dual-energy imaging—the scope of cardiac assessment has expanded beyond the coronary arteries to include valves, myocardium, and the entire heart. This includes evaluating restricted AV opening and cardiac function using four-dimensional imaging, assessing AV annulus diameter and AS severity via calcium scoring with a novel motion correction algorithm, and detecting myocardial damage through late-phase contrast imaging using new reconstruction techniques. In cases of pre-TAVI evaluation or congenital bicuspid valves, CT is also valuable for assessing extracardiac structures, such as access routes and associated congenital heart anomalies. In addition, recent advancements in CT technology have made it possible to significantly reduce radiation exposure during cardiac imaging. CT has become an extremely useful tool for comprehensive cardiac evaluation in patients with aortic stenosis, especially those being considered for surgical treatment.

## 1. Introduction

In aortic valve stenosis (AS), a restricted aortic valve (AV) opening impedes blood ejection from the left ventricle (LV) into the aorta, resulting in a pressure gradient between the two and increased pressure load on the LV [1]. Therefore, AS is a potentially fatal cardiac condition that can lead to heart failure or sudden death if left untreated [2]. The most common cause of AS in younger individuals is congenital anomalies, particularly abnormalities in the number of AV leaflets, such as a bicuspid valve [3]. In the elderly, the predominant cause is atherosclerotic degeneration of the AV, with a higher prevalence among those with risk factors such as hypertension, dyslipidemia, smoking history, and renal impairment [4]. Rheumatic fever, often associated with mitral valve abnormalities, is a common cause in middle-aged individuals; however, its incidence has declined in developed countries due to widespread antibiotic use.

The prevalence of AS has been increasing among the elderly [5,6]. With the advent of transcatheter aortic valve implantation (TAVI), increasing attention has been directed toward the accurate diagnosis and severity assessment of AS in this population. In younger patients, AS often results from congenital conditions such as bicuspid aortic valve, as mentioned above [7]. These congenital anomalies are frequently accompanied by other structural abnormalities, including aortic dilation or intracardiac shunts, necessitating detailed anatomical evaluation in such cases.

Traditionally, the diagnosis and assessment of AS severity have relied primarily on echocardiography [8]. However, emerging imaging modalities such as computed tomography (CT) have recently garnered significant interest in evaluating valvular diseases. This manuscript discusses the evolving role of cardiac CT in the diagnosis of AS, with a particular focus on recent technical advancements in CT imaging for the evaluation of this condition.

## 2. Improvement of the Treatment of Aortic Valve Stenosis

Surgical aortic valve replacement (SAVR) has been a standard treatment for severe aortic stenosis (AS), significantly improving patient prognosis [9,10]. Although some elderly patients were previously left untreated due to the high risk associated with invasive procedures, the advent of transcatheter interventions has greatly expanded the treatable age range.

However, since transcatheter aortic valve implantation (TAVI) is a relatively new treatment, its long-term durability has not been fully established, making it challenging to apply to younger patients. Recently, the option to implant a new TAVI valve within a deteriorated one (valve-in-valve TAVI) has become available, resulting in a trend toward offering TAVI to younger patients than in its early years [7]. Nonetheless, careful consideration is essential when selecting treatment, as data on TAVI durability and long-term prognosis remain insufficient—especially in patients with low surgical risk [11].

The choice of valve type is particularly important in younger patients undergoing surgery. Mechanical valves can generally last a lifetime unless complications such as infection occur, but they require lifelong vitamin K antagonist use, which carries a bleeding risk [12]. Vitamin K antagonists are also teratogenic and contraindicated during pregnancy, requiring additional caution in young female patients.

Conversely, bioprosthetic valves are associated with a risk of structural valve deterioration and often require reoperation within 10 to 15 years [13]. However, due to the increasing availability of TAVI after SAVR, bioprosthetic valves are now being considered in younger patients than previously recommended [14].

With the growing number of treatment options, valve selection should be made based on a comprehensive, lifetime management approach. The Ross procedure, which involves replacing the aortic valve with the patient’s own pulmonary valve, is well-established in pediatric patients and has recently shown promising results in adults as well. It may be a viable option at experienced centers [15].

## 3. Improvement of Aortic Valve Stenosis Evaluation Using Computed Tomography

### 3.1. Utility of Computed Tomography in Aortic Valve Stenosis

Cardiac computed tomography (CT) has become widely used in recent years for coronary artery disease screening in cardiovascular practice [16]. Beyond this application, advances in CT technology have enabled the detection of left ventricular (LV) myocardial fibrosis through late-phase imaging, and functional cardiac analysis across the cardiac cycle with reduced radiation exposure [17].

In addition, four-dimensional imaging allows for motion analysis of the aortic valve. Current guidelines now recommend the use of CT in assessing the severity of valvular disease, particularly through calcification scoring using non-contrast imaging [18].

### 3.2. Utility of the Screening of Coronary Artery Stenosis on Computed Tomography

With appropriate patient selection, coronary CT angiography (CTA) demonstrates high accuracy in detecting significant coronary artery stenosis, particularly due to its high negative predictive value [19]. Therefore, it is useful for screening coronary artery disease in patients with aortic stenosis (AS), especially among the elderly.

In recent years, improvements in temporal resolution have enabled direct evaluation of the aortic valve (AV) itself [20]. Moreover, with the advent of new motion correction algorithms, higher diagnostic accuracy for significant coronary artery stenosis can now be achieved, particularly in patients with elevated heart rates [21]. These algorithms also help reduce motion artifacts in the imaging of the AV.

**Figure 1:**
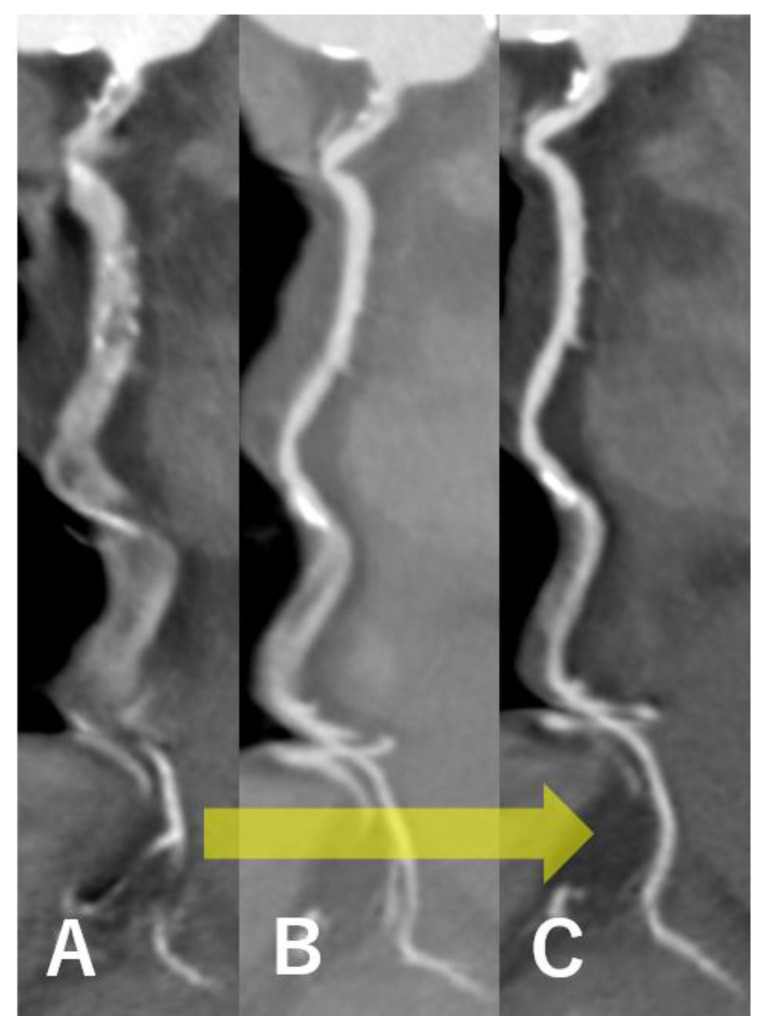
Motion artifact improvement in the right coronary artery using a new motion correction algorithm. From left to right: images without motion correction algorithm (**A**), with the 1st generation motion correction (**B**), and with the 2nd generation motion correction (**C**). Motion artifact is gradually reduced by using the new motion correction algorism (yellow arrow).

Figure 1 shows images of the right coronary artery with severe motion artifacts (Figure 1A), which were progressively reduced using first- (Figure 1B) and second-generation (Figure 1C) motion correction algorithms.

Recent studies have reported that these new algorithms are useful not only for improving the diagnostic accuracy of anatomically significant coronary artery stenosis but also for evaluating functionally significant lesions [22].

Reduction in radiation exposure in cardiac CT is particularly important, especially in younger patients. When cardiac CT was first introduced, its high radiation dose was considered a major concern. However, recent advancements in scanner technology have enabled significant dose reductions during cardiac CT examinations [23].

One of the most impactful innovations has been the introduction of wide-coverage multi-slice detector rows in the longitudinal direction. While this development has primarily been highlighted for its ability to reduce coronary artery “stepping” artifacts in cases with arrhythmia, it also plays a significant role in reducing radiation exposure. This is achieved by eliminating the overlap in scan coverage that occurred with earlier detectors that had fewer rows [23].

Additionally, advances in image reconstruction techniques have made it possible to acquire high-quality images with low image noise—even at low radiation doses. In the past, filtered back projection (FBP) was the only available image reconstruction method for CT. Recently, however, newer techniques such as iterative reconstruction and deep learning reconstruction (DLR) have been introduced. These methods have proven effective in significantly reducing radiation exposure while maintaining high image quality in coronary imaging [24].

In younger patients with AS, repeated CT imaging may be necessary for postoperative follow-up or evaluation of comorbid cardiac and systemic diseases, raising ongoing concerns about radiation exposure. Nevertheless, given the substantial reduction in radiation dose achieved by these newer technologies, comprehensive cardiac evaluation—including preoperative coronary artery screening—can now be considered feasible even in this population.

Noise reduction with these advanced reconstruction techniques is also beneficial for cardiac evaluations such as early-phase imaging for coronary artery assessment in obese patients, and late-phase imaging for detecting myocardial damage, as discussed below.

### 3.3. Computed Tomography for Evaluating the Significance of Aortic Valve Calcification

While the severity of AS is generally assessed by echocardiography, in cases where it is difficult to determine whether surgical treatment is warranted, the aortic valve (AV) calcium score on non-contrast CT can be used as an alternative measure [18].

However, since the AV is a rapidly moving structure, motion artifacts can appear on non-contrast CT images, leading to an overestimation of the calcification score. Moreover, because prospective ECG-gated imaging is typically used to minimize radiation exposure in non-contrast scans, data acquisition is limited to a short phase of the cardiac cycle. This limitation can result in substantial motion artifacts, particularly in patients with high heart rates or arrhythmias.

The latest generation of motion correction algorithms, such as Snapshot Freeze 2, have demonstrated effectiveness not only in coronary artery imaging but also in reducing motion artifacts in AV imaging. This is especially beneficial in challenging cases.

**Figure 2:**
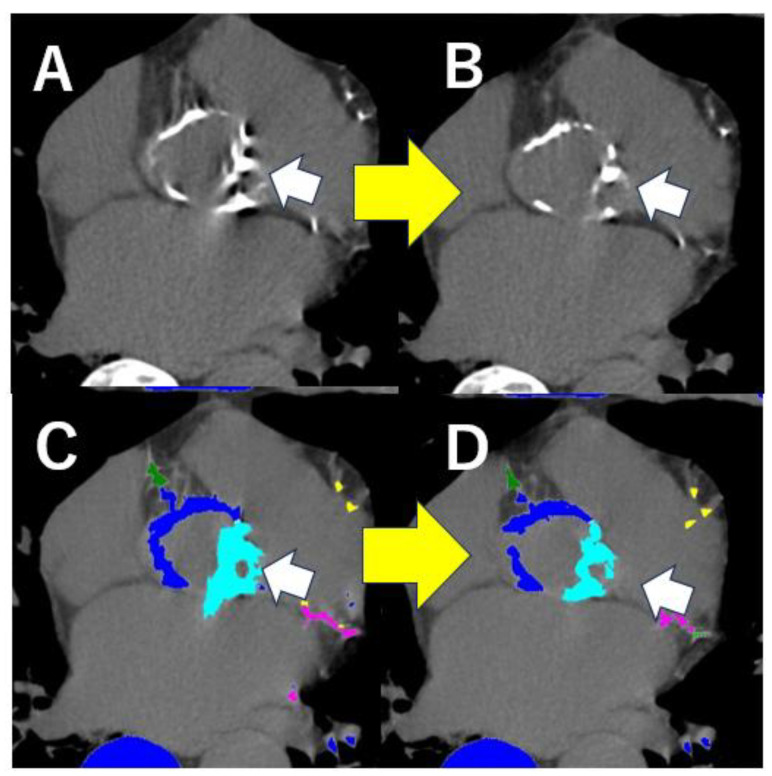
Usefulness of the 2nd generation motion correction algorithm in a case with severe motion artifacts for aortic valve calcification score analysis. Comparison of images of a severely calcified aortic valve (AV) (white arrows) with and without severe motion artifacts is shown. These were calcified AV images from the same patient without the second-generation motion correction algorithm (SSF2) (**A**) and with SSF2 (**B**). The Agatston calcium score of the AV was measured using both images. The score decreased from 2875 (**C**) in the image without SSF2-corrected image to 2214 (**D**) in the image with SSF2, mainly due to the presence of motion artifacts. Using the new motion correction algorism, motion artifact of aortic valve calcification is reduced (upper yellow arrow) and the calcification score is also reduced (lower yellow arrow).

Figure 2 shows a non-contrast CT image from a patient with a heart rate of 110 bpm during the scan. The application of the second-generation motion correction algorithm (Snapshot Freeze 2, GE HealthCare, Waukesha, WI, USA) significantly reduced motion artifacts and resulted in a lower, more accurate calcification score.

**Figure 3:**
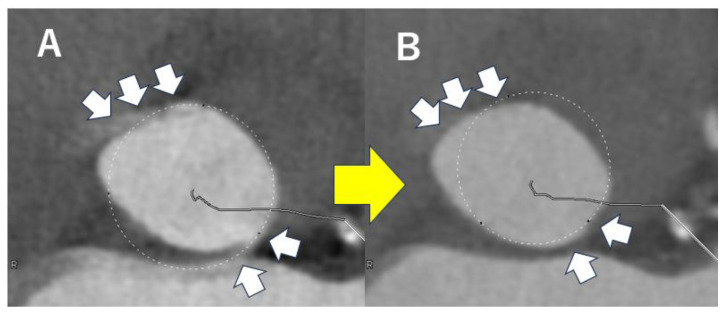
Usefulness of the 2nd generation motion correction algorithm in a case with severe motion artifacts for the measurement of the size of aortic annulus. The size of aortic annulus is usually measured on the systolic image before transcatheter aortic valve implantation (TAVI) at 30% of the R-R waves interval. These are the images of aortic annulus in the same patient, without the 2nd generation motion correction algorithm (**A**) and with (**B**). The 2nd generation motion correction algorithm is useful to decrease the motion artifacts of aortic annulus (white arrows) for the accurate measurement of the size of aortic annulus to decide the artificial valve in TAVI. The new motion correction algorism reduces motion artifact-induced blurring of the aortic valve annulus (yellow arrow).

This motion correction algorithm is also useful for the accurate measurement of the size of the aortic annulus, especially in cases with higher heart rate and severe motion artifacts just before the aortic valve surgery (Figure 3), but the details of its measurement will be discussed in detail in Section 4.

### 3.4. Utility of the Evaluation of Congenital Abnormalities on Computed Tomography

CT is valuable for evaluating the internal structure of the heart in three dimensions, and it is useful not only for assessing valvular disease but also for identifying congenital abnormalities such as intracardiac shunts [25].

**Figure 4:**
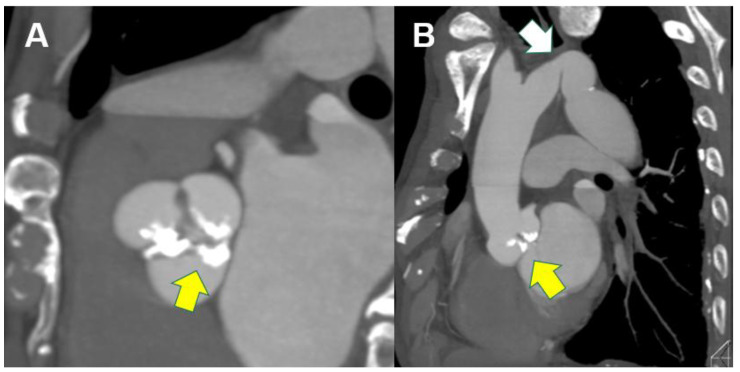
Thoracic aortic coarctation in a case with severe aortic valve stenosis. These were maximum-intensity projection images of computed tomography in a case with severe aortic valve stenosis and aortic coarctation. Panel (**A**) shows severe aortic valve calcifications on a short axial image of the aortic valve. Panel (**B**) shows coarctation of the aortic arch and severe aortic valve calcifications. Yellow arrows indicate a highly calcified aortic valve. A white arrow indicates aortic coarctation.

In particular, in young patients with aortic stenosis (AS), it is important to assess not only valve abnormalities but also complex congenital heart malformations. In such cases, CT serves as a useful adjunct to echocardiography. As mentioned above, AS caused by bicuspid aortic valves is common in younger patients. CT is useful in evaluating the aortic valve leaflets (Figure 4A), which also allows the classification of bicuspid valves. The Sievers classification is the most widely used classification of bicuspid valves. The Sievers classification is based on the valvular leaflet fusion pattern and the presence or absence of a raphe. Type 0 is a true bicuspid valve with no commissure raphe, Type 1 is a bicuspid valve with one commissure raphe, and Type 2 is a bicuspid valve with two commissure raphes (equivalent to a unicuspid valve). Type 1 is further subdivided according to the combination of the fused valve leaflets [26].

In these individuals, ascending aortic enlargement is frequently observed as a comorbidity [3]. Therefore, CT is beneficial for evaluating aortic abnormalities, the aortic valve, and coronary artery stenosis—especially in the preoperative setting. Aortic coarctation is a known congenital condition that is often associated with bicuspid aortic valves and subsequent aortic stenosis (Figure 4B). CT is particularly useful for comprehensive evaluation in such cases with severe AS [27].

### 3.5. The Analysis of Myocardial Damage on Computed Tomography

In recent years, cardiac CT has been used in some clinical research to detect myocardial fibrosis as late-enhanced lesions by adding a late-phase scan a few minutes after the early contrast phase of coronary artery imaging [28]. Although this delayed contrast imaging has been technically feasible with CT, concerns about image quality and additional radiation exposure have limited its clinical use, giving way to magnetic resonance imaging (MRI) until recently. However, with recent advancements in cardiac CT—specifically lower radiation doses and improved image quality in the late contrast-enhanced phase—its use in routine clinical practice is becoming more feasible. Using the latest equipment, the detectability of left ventricular (LV) late enhancement by CT is now considered to be comparable to that of MRI [29].

**Figure 5:**
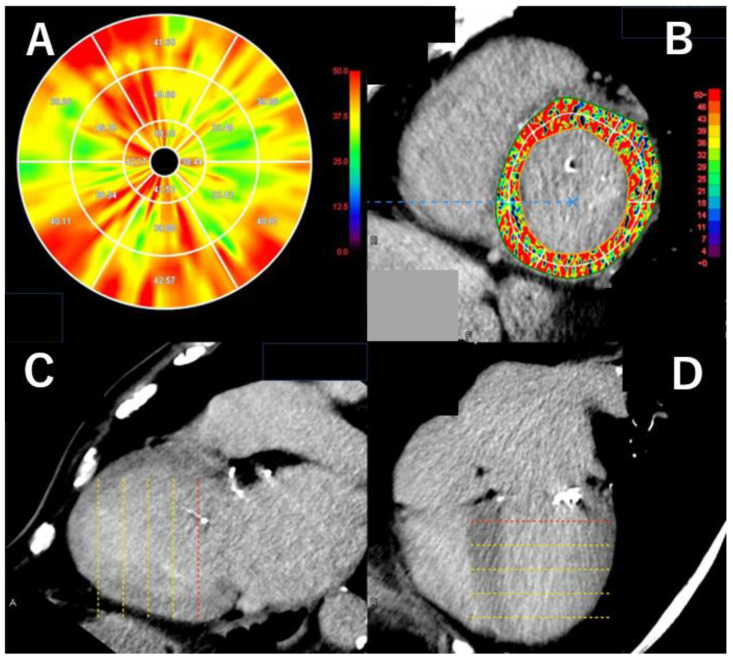
Extracellular volume analysis of left ventricular myocardium in a case with severe aortic valve stenosis. Extracellular volume (ECV) of left ventricular myocardium (LVM) on computed tomography (CT) in this patient with severe aortic valve (AV) stenosis was 39.37% (**A**), and this patient was admitted because of congestive heart failure 12 months after the transcatheter AV implantation(TAVI). ECV was measured using a specific software (Ziostation 2, Ziosoft, Tokyo, Japan). Short axial, 2-chamber, and 4-chamber views of late enhancement LVM were shown in Figure (**B**–**D**). The dotted lines over the LV in the long-axis images (**C**,**D**), indicate the slices of the short-axial images used for analysis (The red dotted line indicates the currently displayed short axis image) (**B**).

Evaluation of LV late enhancement has originally been useful for differentiating underlying myocardial diseases based on enhancement patterns, and the use of modern CT scanners has proven effective in this differentiation as well [30]. Recently, not only qualitative but also quantitative assessment of late enhancement has become possible with the development of new image analysis software, such as left ventricular extracellular volume (LV-ECV) quantification (Figure 5).

LV-ECV assessment was first made possible by the advent of T1 mapping in MRI. It is known to correlate well with the histopathological burden of myocardial fibrosis in dilated cardiomyopathy based on biopsy findings and is regarded as a safe and minimally invasive surrogate marker for pathological evaluation [31]. CT-based evaluation of LV-ECV before surgery for aortic stenosis (AS) has been reported to be useful in predicting the risk of postoperative cardiac events [32]. Furthermore, recent studies have shown that CT-based LV-ECV analysis before TAVI can help predict postoperative prognosis.

Moreover, it is known that a certain percentage of patients undergoing TAVI have cardiac amyloidosis, and CT-based LV-ECV evaluation can be very useful for detecting such cases [33]. With recent advances in the treatment of cardiac amyloidosis, timely detection and diagnosis have become increasingly important. Current guidelines suggest that delayed contrast-enhanced CT imaging may be useful for detecting cardiac amyloidosis when MRI is not feasible, and its clinical adoption is expected to grow [34].

However, it should be noted that the image quality of LV late enhancement in CT correlates positively with the amount of contrast agent administered and negatively with the patient’s body mass index (BMI). Therefore, image quality may deteriorate in patients who require contrast dose reduction due to renal impairment or in those who are obese, and caution should be exercised in such cases [17].

### 3.6. Myocardial Strain Analysis on Computed Tomography

In cardiac CT imaging for coronary artery evaluation, prospective electrocardiography (ECG) gating has been used to minimize radiation exposure by performing short-duration scans, typically only during the diastolic phase, which is generally considered to have the least cardiac motion artifacts [23]. However, with the advent of wide-coverage scanners and other technological advancements that have enabled further radiation dose reduction, it has recently become feasible to analyze cardiac function by scanning the entire cardiac cycle using CT. Numerous studies have been published employing this protocol.

**Figure 6:**
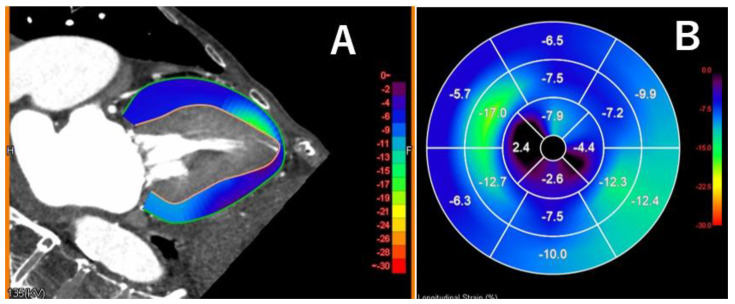
Myocardial longitudinal strain analysis in a patient with severe aortic stenosis. Myocardial longitudinal strain (LS) analysis was performed using specific software (Ziostation REVORAS v5.3.0.0, Ziosoft, Tokyo, Japan) in a case with severe aortic stenosis. Peak global LS of the left ventricular myocardium was measured using longitudinal views (Panel **A**) and decreased to −7.4 (Panel **B**), and this patient was admitted because of congestive heart failure 13 months after the transcatheter aortic valve replacement.

In particular, cardiac CT performed prior to TAVI typically evaluates the aortic valve complex during systole and the coronary arteries during diastole, which usually requires image acquisition throughout one full cardiac cycle. Several studies have reported cardiac functional assessments using this approach [35]. Recently, advances in image analysis software have enabled not only the measurement of left and right ventricular ejection fraction but also myocardial strain analysis [35]. Figure 6 illustrates LV strain analysis using CT and a dedicated software tool in a patient with severe AS. The CT scan was acquired immediately prior to TAVI. Global longitudinal strain (GLS) was markedly reduced to −7.4%. The patient was hospitalized for heart failure 13 months after the TAVI procedure.

## 4. Utility of CT in Patients with AS Before and After Invasive Procedures

### 4.1. General Utility of CT for AS Before Invasive Procedures

Preoperative assessment of the structures surrounding the aortic valve (AV) prior to surgical aortic valve replacement (SAVR) or TAVI is crucial to ensure appropriate treatment. In cases of severe aortic stenosis (AS) with concomitant aortic root or ascending aortic dilation, simultaneous surgical correction may be considered. The size of the AV annulus and the distribution of calcification around it are important factors in selecting the appropriate size and type of prosthetic valve. For patients with a small annulus, the need for annular enlargement procedures can be evaluated preoperatively.

### 4.2. Analysis of Aortic Valve Complex on CT Before TAVI

In TAVI, intraoperative measurement of the AV annulus is not feasible; thus, highly accurate preoperative assessment is essential to avoid serious complications such as annular rupture or postoperative aortic regurgitation, the latter of which is associated with poor prognosis.

Traditionally, measurement of the AV annulus diameter has been performed using angiography or echocardiography, but inconsistencies among these modalities have been a clinical issue [36]. This limitation is largely due to the two-dimensional nature of these methods, whereas the AV annulus is not perfectly circular but rather oval-shaped, making computed tomography (CT) a valuable tool for evaluation.

Using ECG-gated CT images acquired during the systolic phase, a cross-sectional view of the valvular ring is obtained by identifying the lowest (hinge) points of the right coronary, left coronary, and non-coronary cusps. The virtual annular plane connecting these three hinge points is referred to as the virtual basal ring [37]. Valve sizing and calcification are then assessed based on this virtual annulus. Typically, valve size selection is based on the annular area for balloon-expandable TAVI valves and on the annular perimeter for self-expanding valves.

Especially in bicuspid valves, it is necessary to evaluate the presence or absence of Raphe and the intercommissural distance. The latter is usually measured at a position such as 4 mm above the annulus. The latter is an indicator of the space in which the prosthetic valve is actually deployed. Especially in BAVs, this distance may have more influence on valve sizing than the annulus [38].

This is a typical image of an 89-year-old female with severe ubiquitous aortic valve annulus calcification on computed tomography which was performed just before transcatheter aortic valve replacement. She also has heavy calcification of the left ventricular outflow tract, which places her at high risk of annular rupture with balloon-expandable valves and severe paravalvular leak with self-expanding valves.

Measure the size of the right and left coronary cusps individually and assess their degree of calcification. In cases with a small sinus of Valsalva (SOV), the risk of coronary occlusion or rupture of the SOV is high. In patients with bicuspid valves, calcification of the raphe should be carefully evaluated, and valve sizing should take into account the possibility that the raphe may not be mobile.

Balloon aortic valvuloplasty should also be considered during TAVI, but caution is necessary in cases with a small sinotubular (ST) junction, as there is a risk of aortic injury caused by contact between the balloon shoulder and the aortic wall outside the stent frame of a balloon-expandable TAVI valve [39]. Additionally, particular caution should be taken in cases with severe calcification. It should be noted that the ST junction, along with the valve annulus size, can be a determining factor in selecting the appropriate prosthetic valve.

**Figure 7:**
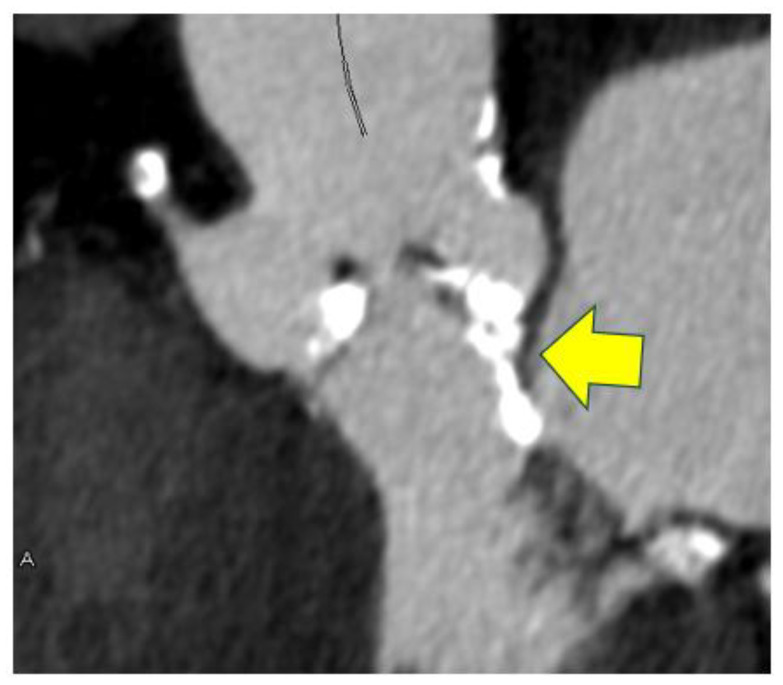
Severe calcification of aortic annulus in a case with severe aortic stenosis. An yellow arrow indicates severe calcification contiguous from the aortic annulus to the lower part of the aortic valve.

Evaluation of the left ventricular outflow tract (LVOT) is also important to avoid annular rupture during TAVI. In a review of valve rupture cases, continuous calcification from the valve annulus to the LVOT was observed (Figure 7), with the calcification often located on the epicardial fat side [40]. Because annular rupture has a very poor prognosis, preoperative planning is essential—for example, choosing a self-expanding valve for patients at high risk as assessed by CT. Regarding coronary ostial height, it has been reported that the risk of coronary occlusion increases when the height is less than 10–12 mm [41].

### 4.3. Analysis of Catheter Access Route on CT Before TAVI

Assessment of calcification distribution, the presence of stenosis, or tortuosity throughout the entire vascular route—from both common femoral arteries to the ascending aorta—is crucial for determining the most appropriate vascular access route for TAVI, or for placing percutaneous cardiopulmonary support or other circulatory devices in case of sudden hemodynamic deterioration. Although the transfemoral approach is commonly used for TAVI, the trans-subclavian approach may be necessary when femoral access is deemed difficult; thus, evaluation of all access vessels is important.

Since accurate evaluation of AS severity may be difficult in cases of low-pressure gradient, etc., it is expected that scoring to appropriately diagnose severe AS, such as scoring to predict the onset of atrial fibrillation or left ventricular diastolic dysfunction [42,43], will be constructed.

### 4.4. CT Analysis Before Valve-In Valve TAVI or TAV in TAV

Advances in TAVI therapy have made it possible to perform procedures even in patients with prior surgical bioprosthetic valve replacement or prior TAVI, which were previously considered challenging. Valve-in-valve and TAV in TAV are known to have a higher risk of coronary artery occlusion; therefore, special attention is required in these cases, as they necessitate a different preoperative assessment compared to native valve cases [44].

In valve-in-valve procedures, annular sizing is based on the most basal points of the surgical bioprosthetic valve basal ring, rather than the native anatomical annulus. In TAV-in-TAV procedures, sizing should be based on the inner diameter of the stent frame of the first transcatheter heart valve (THV). It is also crucial to evaluate the inner frame diameter (inflow area), frame height, leaflet mobility, presence of calcification, and pannus formation. Additionally, the idea of estimating the distance between the anticipated final position of the expanded THV frame and the coronary artery orifice has been defined as the virtual THV-to-coronary (VTC) distance [45]. According to a recent multicenter analysis, a VTC of less than 4 mm has been identified as the sole independent predictor of coronary obstruction [46].

### 4.5. Pulmonary Assessment on CT Before TAVI

Additionally, pulmonary assessment is vital in the preoperative evaluation to determine the level of invasiveness and the appropriate anesthesia method. According to Japanese guidelines for valvular heart disease, TAVI should be considered in patients with severe AS and comorbid obstructive pulmonary disease or interstitial pneumonia [18]. Because of the risks associated with general anesthesia and surgical invasiveness, performing TAVI under local anesthesia can be advantageous. Patients undergoing TAVI are often elderly, and extracardiac abnormalities are sometimes incidentally detected—malignancies are found on preoperative TAVI CT in approximately 4% of cases [47]. In such instances, non-cardiac diseases may significantly impact prognosis, requiring careful assessment when determining TAVI eligibility. A comprehensive evaluation that includes extracardiac conditions is therefore essential.

### 4.6. CT Analysis After TAVI

Contrast-enhanced CT performed after TAVI shows a hypo-attenuated area above the valve leaflet in approximately 10–15% of cases, referred to as hypo-attenuated leaflet thickening (HALT) [48]. This finding is often considered thrombotic in nature and tends to resolve with anticoagulant therapy. However, the clinical significance of HALT, including its impact on valve function, durability, and long-term outcomes, remains unclear. The optimal antithrombotic strategy, treatment indications, and therapeutic interventions for HALT after TAVI have yet to be established.

### 4.7. Blood Flow Analysis Using CT

**Figure 8:**
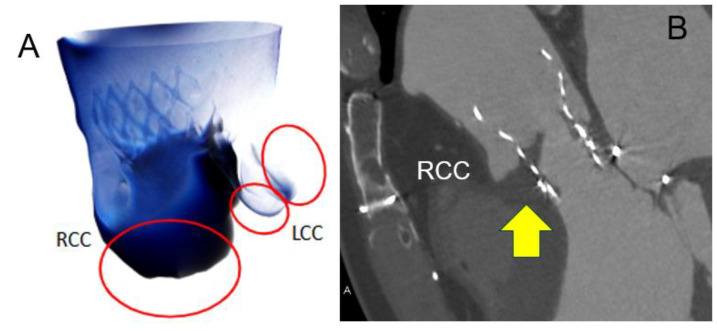
Compute tomography blood flow analysis around sinus of Valsalva in a case after transcatheter aortic valve replacement. (**A**): The computed tomography (CT) flow analysis of this case after transcatheter aortic valve replacement shows a marked decrease in blood flow (dark blue lesion) in the right coronary cusp (RCC) of sinus of Valsalva (SOV) (red circles mean each cusp of SOV, LCC means left coronary cusp). (**B**): A huge thrombus was detected in the RCC of SOV on CT in the same patient (yellow arrow).

Recently, it has become possible to perform blood flow analysis using computational fluid dynamics based on cardiac CT images [49]. Figure 8 shows a case of a thrombosed prosthetic valve in the sinus of Valsalva (SOV) after TAVI. In this case, CT-based flow analysis revealed markedly reduced blood flow in the right coronary cusp region of the SOV. Such CT flow analysis is expected to become a valuable tool in assessing the risk of prosthetic valve thrombosis after TAVI.

## 5. Summary of Utility of CT for Patients with AS

As mentioned earlier, cardiac CT is very useful for comprehensive cardiac evaluation in AS. In patients with severe AS, CT is useful for preoperative coronary assessment and for evaluating the aortic valve complex, including the annulus and leaflets—particularly in cases where transthoracic echocardiography is inconclusive. In patients with bicuspid aortic valves, CT also enables the assessment of concomitant cardiac malformations and aortic abnormalities, and such vascular evaluation remains useful even prior to TAVI.

Moreover, late enhancement imaging for detecting myocardial damage and four-dimensional CT for assessing cardiac function—techniques previously limited—are expected to contribute to prognostic evaluation. Evaluation of aortic valve calcification is particularly valuable in AS cases where severity cannot be accurately assessed by echocardiography. In addition, CT allows for the evaluation of extracardiac comorbidities, such as pulmonary disease, which may influence surgical risk.

**Figure 9:**
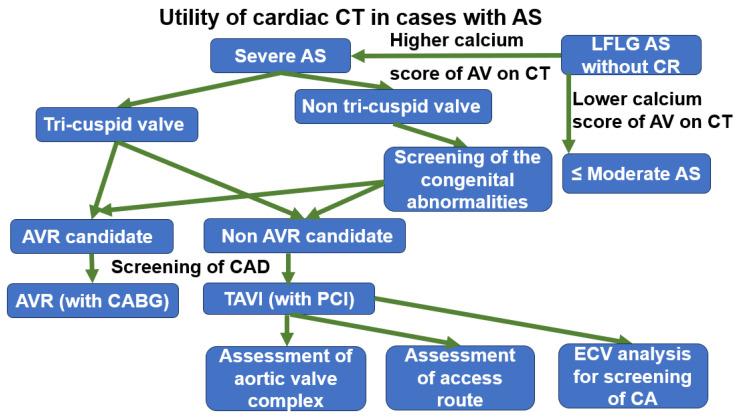
Summary of utility of cardiac computed tomography for evaluation of aortic valve stenosis. AS: aortic valve stenosis, AV: aortic valve, LFLG: low flow low gradient, CR: contractile reserve, CT: computed tomography, AVR: aortic valve replacement, TAVI: transcatheter aortic valve implantation, CAD: coronary artery disease, PCI: percutaneous coronary artery intervention, CABG: coronary artery bypass grafting, CA: cardiac amyloidosis.

Recent advances in CT technology have enabled these comprehensive assessments to be performed with high accuracy and reduced radiation exposure, and further improvements are anticipated. Figure 9 summarizes the main aspects discussed.

## 6. Conclusions

Cardiac CT has become very useful in the comprehensive cardiac evaluation of AS in various age groups due to its technological innovations. Clinicians are required to fully understand and clinically use it in cardiovascular practice.

## Data Availability

No data from this article will be shared.

## Abbreviations

The following abbreviations are used in this manuscript:

AS	Aortic valve stenosis
AV	Aortic valve
HALT	Hypo-attenuated leaflet thickening;
AVR	Aortic valve replacement
TVAR	transcatheter aortic valve replacement;
IVC	inferior vena cava;
CRRT	continuous renal replacement therapy;
CT	Computed tomography;
ECV	Electrocardiography
MRI	Magnetic resonance imaging;
ECV	Extracellular volume;
DLR	Deep learning reconstruction
